# Effectiveness of virtual reality glasses with integrated sign language in reducing dental anxiety during pulpotomy in children with hearing impairment: a randomized controlled trial

**DOI:** 10.1186/s12903-024-05129-1

**Published:** 2024-11-15

**Authors:** Reem Moustafa Salama, Laila Moustafa El-Habashy, Sarah Ibrahim Zeitoun

**Affiliations:** 1 Pediatric Dentistry and Dental Public Health Department, Faculty of Dentistry, Alexandria University, Champollion St., Azarita, Alexandria, 21527 Egypt; 2 Pediatric Dentistry Department, Faculty of Dentistry, PUA University, Alexandria, Egypt

**Keywords:** Hearing impairment, Dental anxiety, Distraction, Virtual reality

## Abstract

**Background:**

Children with hearing impairment (HI) face communication challenges during dental procedures due to hearing loss. Studies suggest that distraction techniques, like virtual reality (VR), can effectively divert their focus from stressful stimuli, resulting in a more comfortable dental experience. The present study aims to assess the effectiveness of distracting children with moderate to severe (HI) with virtual reality glasses that show cartoons in sign language during pulpotomy treatment compared to conventional management techniques.

**Methods:**

Forty children aged five to seven participated in a randomized controlled parallel two-arm clinical trial—the type of behavioral management employed determined which two groups children were randomly placed into. Group I (Study group) used virtual reality glasses as a diversion, while Group II (Control group) used the conventional behavior management approach. Local anesthesia was administrated, and a pulpotomy procedure was performed on the selected tooth, followed by stainless steel crown restoration (SSC). There were three methods used to assess dental anxiety before and after the procedure: the physiological method, which used heart rate (HR); the objective measure, which used the Venham Clinical Anxiety Scale (VCAS); and the subjective measure, which used the modified Facial Affective Scale (FAS). An independent t-test was employed for HR analysis of the difference between the groups as a continuous variable. The Pearson Chi-square test assessed differences between groups for categorical variables, such as (VCAS) (FAS).

**Results:**

No significant differences were found in mean (HR) or (VCAS) between the two groups throughout the procedures: during local anesthesia (*p* = 0.659, 0.282), pulpotomy (*p* = 0.482, 0.451), and stainless steel crown preparation (*p* = 0.090, 0.284). Anxiety levels by (FAS) remained statistically comparable between the two groups before and after the procedures (*p* = 0.507, 0.749), respectively.

**Conclusions:**

The use of VR glasses revealed no significant advantages in managing children with HI during the dental visit compared to the conventional method of child behavior management.

**Trial registration:**

The trial was prospectively enrolled on 11/11/2023 under the identification number NCT06153823 on ClinicalTrials.gov.

**Supplementary Information:**

The online version contains supplementary material available at 10.1186/s12903-024-05129-1.

## Background

Pediatric patients frequently exhibit anxiety during medical interventions, particularly in dentistry. This dental anxiety, characterized by an irrational fear of dental procedures and visits, significantly hinders treatment [1]. Dentists may require additional time to establish trust and implement anxiety-reduction techniques for effective patient management [2].

Hearing impairment (HI) is a decreased ability to hear due to physical damage or abnormalities in the auditory system’s physical structure or function. It can range from mild to profound and affects one or both ears [3]. The severity of hearing loss determines the quality and quantity of the child’s hearing ability and the preferred method of communication [4].

HI hinders speech-language development in children, impacting their overall cognitive, social, and intellectual skills [5]. This highlights the importance of clear communication strategies for children with HI in dental settings. Sign language is also a communication method used by children with HI. It is a face, hands, and arm virtual gesture language. It is similar to oral language in phonology, morphology, syntax, and grammar [6].

The dentist should explain everything that happens because the patient with HI fears the unknown. Show-tell-do (STD) should always be the order in which tasks are performed. When presenting to a deaf patient, body language and facial expressions are crucial. As such, it is best to avoid wearing a mask when providing directions to the patient, as this will obscure most of their facial expressions and make it difficult for them to see gestures and lip reading [7].

In recent years, dental practitioners have tried several methods to distract a child from the dental environment. Audiovisual distraction has effectively reduced dental anxiety in normal children [8]. Virtual reality glasses (VR), a computer-generated, modified three-dimensional (3D) environment, is one of the audiovisual distraction methods. It creates an immersive 3D world that blocks a child’s sight and sound during dental visits. This virtual world keeps them entertained and distracted from the sights and sounds of the dentist’s office, potentially reducing anxiety and making the experience more positive [9].

Few researchers have investigated whether VR could calm anxious children with HI during dental visits. However, the results were contradictory: Kaur et al. [10] and Varshitha et al. [11] found that VR distraction significantly lowered anxiety in children with HI compared to other methods, suggesting VR is a helpful tool. On the other hand, Fakhruddin et al. [12] showed that VR glasses increased anxiety; thus, VR’s effectiveness might not be universal.

Regrettably, the effectiveness of virtual reality glasses in lowering dental anxiety in children with HI has not been sufficiently studied. Therefore, this study aims to investigate whether VR glasses displaying cartoons in sign language could benefit children with moderate to severe HI by distracting them and lessening their anxiety during pulpotomy treatment.

The study’s null hypothesis proposed that there would be no significant difference in the dental anxiety experienced by children with HI during pulpotomy treatment regardless of whether they were managed by VR glasses that displayed cartoons in sign language or the conventional methods of child behavior management techniques.

## Methods

### Ethical consideration

The study was approved by Alexandria University’s Research Ethics Committee (0665-04/2023) and registered on ClinicalTrials.gov (NCT06153823). Informed consent was obtained from parents/guardians after explaining the benefits and risks. The research adhered to the Helsinki Declaration [13] and ethical standards set by the Committee.

### Study design

The research was a two-arm, parallel, randomized controlled clinical trial with a 1:1 allocation ratio. It adhered to the CONSORT [14] organization and documentation requirements and included a completed CONSORT checklist as an additional file. The PICO question was: Do pediatric patients with moderate-severe hearing impairment aged from 5 to 7 years (Population; P) assigned to receive virtual reality glasses distraction (Intervention; I) in comparison to traditional behavior management techniques: STD technique and positive reinforcement (Control; C) exhibit less anxiety during pulpotomy in the primary molar teeth (Outcome; O)?

### Study setting and location

The research was carried out in the Department of Paediatric Dentistry and Dental Public Health, Faculty of Dentistry, Alexandria University, Egypt.

### Sample size estimation

The sample size was computed based on the assumptions of 80% study power and 5% alpha error. The patients who got visual distraction with VR glasses scored 2.25 (0.89) on the mean (SD) anxiety scale during therapy, compared to 3.50 (1.31) for the controls [10], the smallest sample size of 19–20 patients per group, was determined by utilizing the highest SD = 1.31 to provide sufficient power in the difference between independent means. The total sample is 20 × 2 = 40 patients (number per group x number of groups. G*Power 3.1.9.7 determined the sample size using Rosner’s approach [15] as a basis [16].

### Inclusion criteria

A total of 40 children, aged 5–7 years old, with moderate to severe HI and who needed pulpotomy in one of their primary molars, were selected from auditory centers and special hearing impairment schools. The children were selected to show negative to positive behavior with rating scores of 2 or 3 according to the Frankl rating scale [17]. All Parents who agreed to provide permission and take part in the research. Children with any medical conditions beyond hearing impairment (ASA classifications II, III, and IV) [18], with syndromic HI, or those with prior negative dental experiences were excluded.

### Randomization and allocation concealment

Using a computer-generated list of random numbers, participants who met the inclusion requirements were randomized to one of the two arms in a parallel group.

#### Group I

(study group *n* = 20) was assigned to VR glasses distraction with a sign language cartoon.

#### Group II

(control group *n* = 20) was assigned to conventional STD technique and positive reinforcement.

#### Allocation concealment

Unique codes were assigned to each child, along with their group designation. These codes were placed in sealed envelopes containing the corresponding child’s name [19]. Independent of outcome evaluation, a designated individual maintained control of these envelopes, only revealing the code-group link during the local anesthetic injection session to hide the child’s group assignment from the outcome evaluator.

### Blinding

The study used a single-blinded design. The children were unaware of their group assignment, but both the children and the dentist were aware of the intervention received. The statistician analyzing the data was blinded to the group assignments to maintain objectivity. Thus upholding the single-blinded design.

### First dental visit

The first dental visit was a preliminary screening visit, which focused on gently introducing dentistry to the child. A full medical and dental history was taken of each patient. A comprehensive evaluation included a thorough clinical examination and an intraoral periapical radiograph of the designated tooth for restoration. This detailed assessment guaranteed that each participant met the established inclusion criteria.

The study included participants with cochlear implants. These implanted electronic devices restore auditory function in individuals with profound deafness or severe hearing loss. Specific considerations were necessary within a dental setting. During radiographic examinations, the speech processor was positioned at least fifty centimeters from the X-ray source, preferably outside the room [20].

Before treatment, all children were familiarized with the dental equipment and instruments using a modified “STD” technique, which is a modification designed specifically for children with HI. This involved clearer lip-reading, explaining equipment sensations, removing the mask as needed, and directly engaging the child with parental support for sign language interpretation if needed [21]. No treatment was done to the child except for dental prophylaxis to establish a solid patient-dentist bond.

### Second intervention visit

During the second visit, all procedures were also introduced to the child using the STD technique.

#### VR group (study group)

**Fig. 1:**
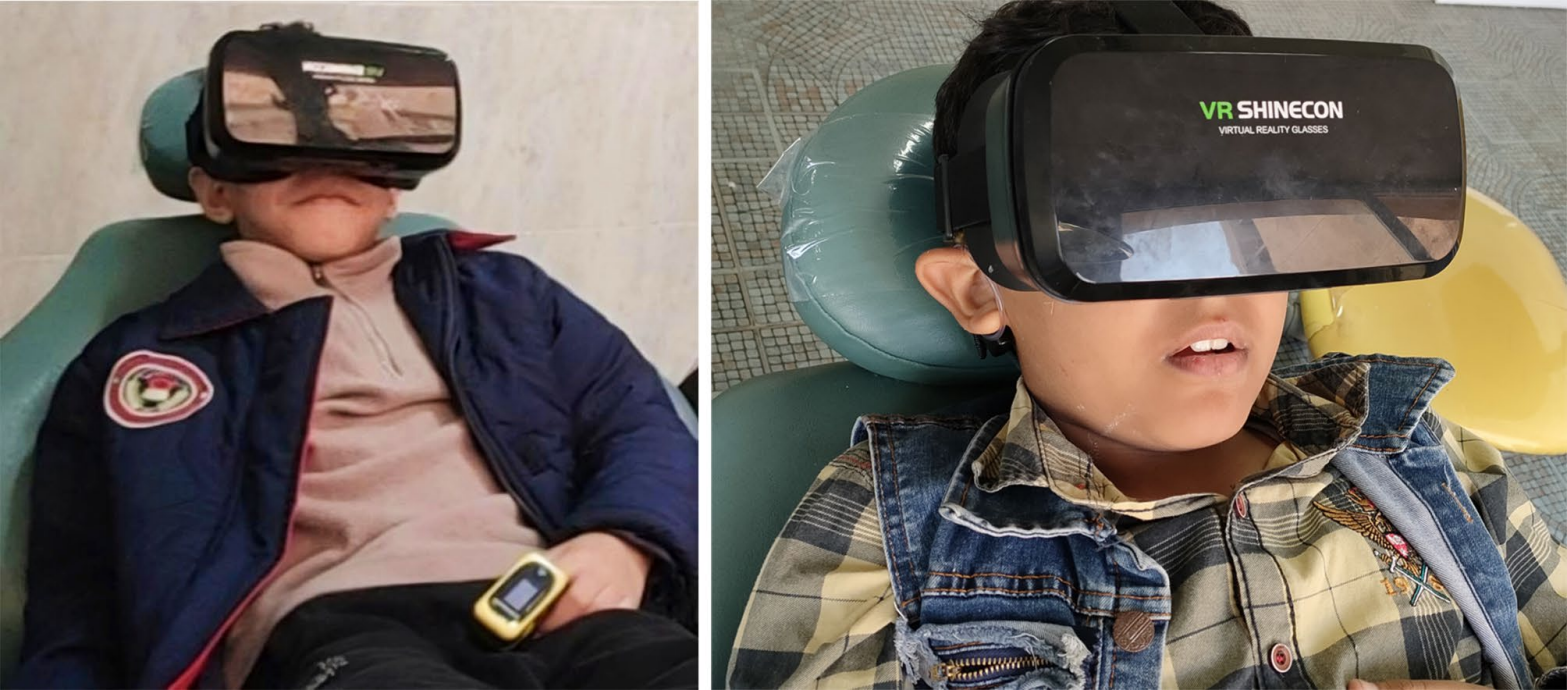
A child with hearing impairment tried the VR glasses before initiating the treatment

The child with HI was given VR glasses (SHINECON VR, china) and allowed to choose his / her favorite cartoon from a list of movies downloaded in sign language. The glasses were adjusted for comfort, and the child was allowed five minutes to get accustomed to them before the procedure began. (Fig. 1)

#### Conventional behavior management group (control group)

The child’s anxiety was reduced by using traditional behavior management strategies such as the STD method and positive reinforcement, along with all the management modifications made for children with HI, as mentioned before, including full visibility of the surroundings [22].

### Intervention

**Fig. 2:**
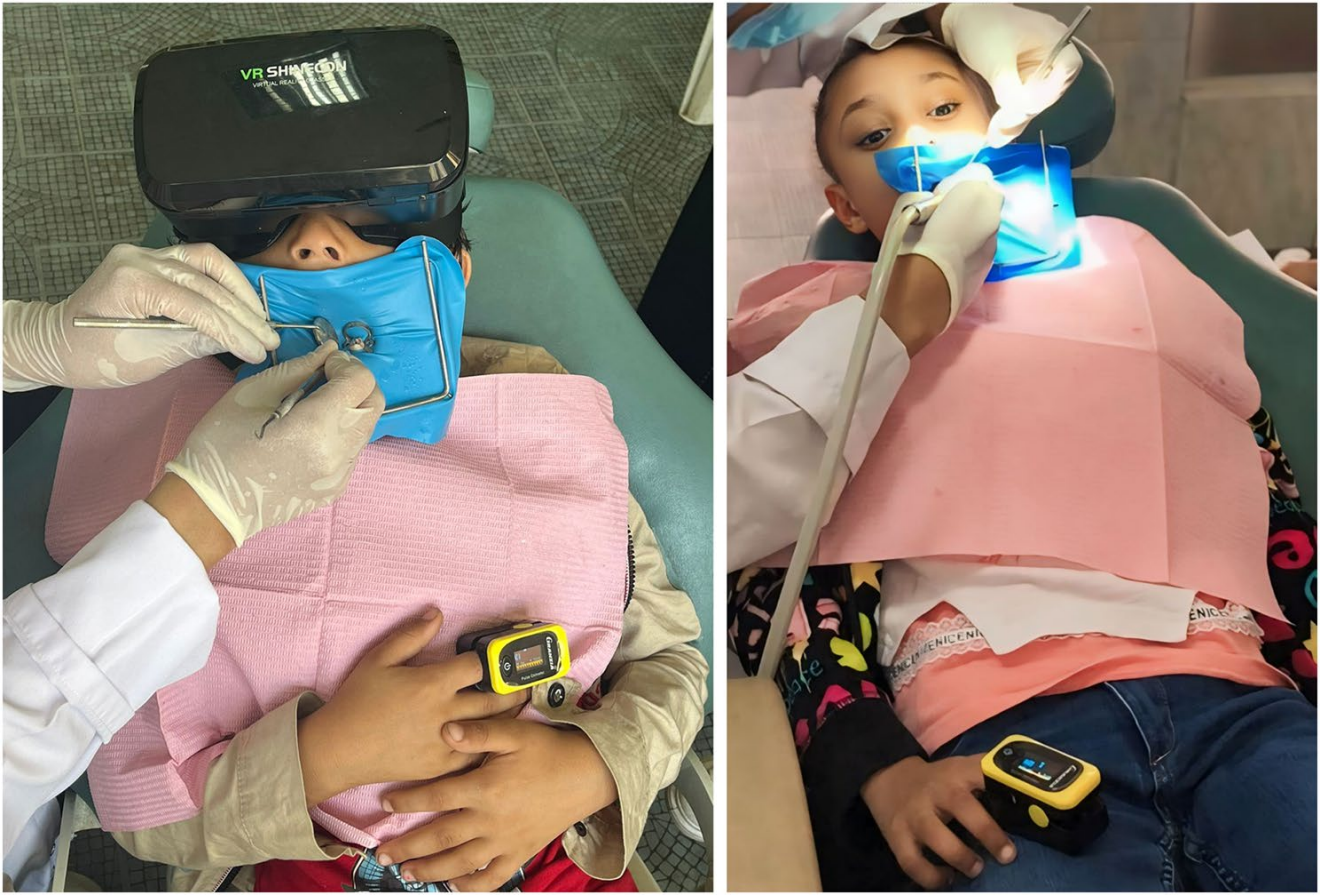
Dental procedure in both study and control groups

In each child in both groups, the MTA Pulpotomy procedure was done in one of their primary molars under complete isolation using a rubber dam according to AAPD guidelines [23], after which SSC was used to restore the tooth [24]. (Fig. 2). The same operator performed all the dental treatments and videotaped dental visits. The injection site was prepared with a topical anesthetic gel (20% benzocaine) (Dharma Research, Inc. 5220 NW 72nd Ave Miami, FL USA) and allowed to numb the area for one minute. An inferior alveolar nerve block needle (C-K Ject, CK Dental Ind. Co., LTD., Korea) was administered for mandibular primary molars. A local anesthetic (Alexandria Co. For Pharmaceuticals, Egypt) was injected into the muccobuccal fold for maxillary primary molars. The effectiveness of the anesthesia was tested by gently probing the gingival tissue. Rubber dams (Waldent Rubber Dam Kit) were used to isolate the teeth, and the caries and coronal pulp chamber roof were removed using a high-speed #330 carbide bur. (Meisinger Carbide Bur-High Speed, Germany). The coronal pulp tissue was carefully removed using a clean, sharp spoon excavator—disinfection of the pulp chamber with 3–5% sodium hypochlorite. Pulpal hemorrhage was controlled by using a sterile, moist cotton pellet under pressure. A paste of MTA was packed gently over the pulp stumps. Finally, all teeth were restored with glass ionomer cement, and SSC (Kids crown Stainless steel) was placed.

### Outcome assessment

**Fig. 3:**
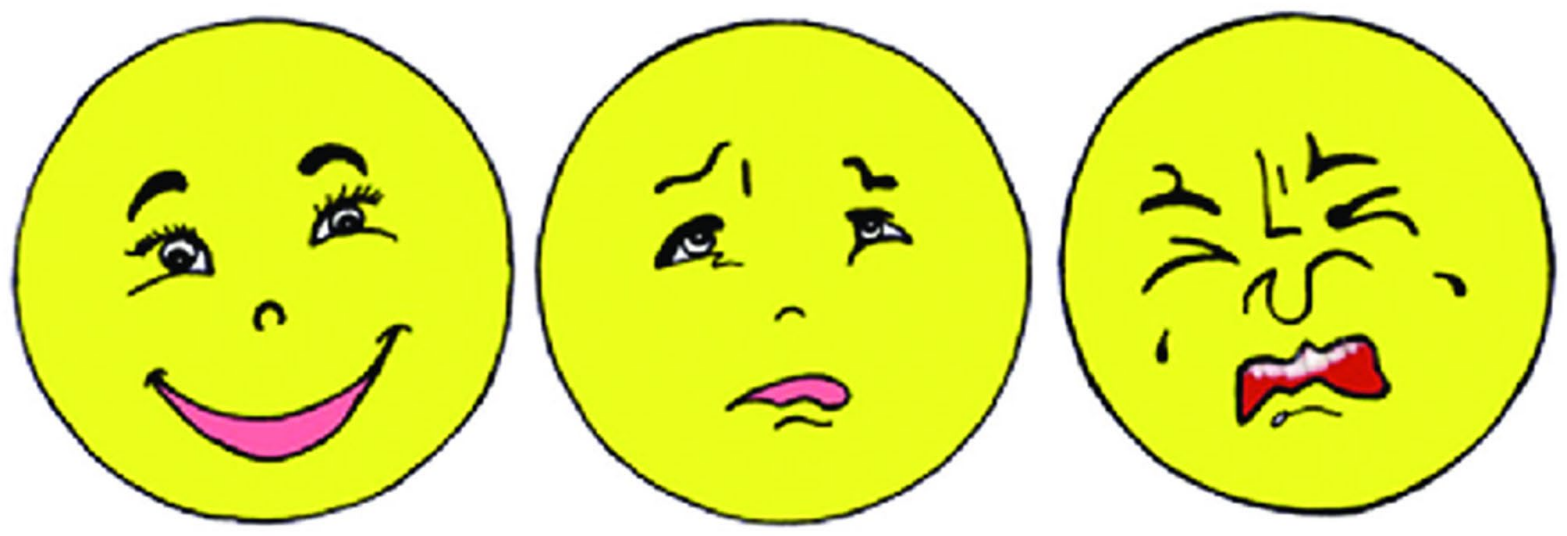
Colored Version of Modified Facial Affective Scale -three faces: (1) No Anxiety; (2) Some Anxiety; (3) Very High Anxiety

The present study used three criteria to assess anxiety: (1) Heart rate as a ***physiological parameter.*** The patient had a brief demonstration of the pulse oximeter’s use, after which it was put on the index finger. Four time points were measured: baseline measurements before local anesthesia (LA), during injection, pulpotomy, and SSC operation. Every two minutes, data were gathered, and an estimate of the mean value was made. **(2)*Objective parameter***: utilizing (the Venham Clinical Anxiety Rating Scale): It rates the child’s anxiety level and provides a behavior description that correlates. It consists of 6 categories (range from 0 to 5) where 0 = relaxed, 1 = uneasy, 2 = tense, 3 = reluctant, 4 = interference, 5 = out of contact. Each category describes the patient’s status in the dental chair when a dental procedure was performed. The assessment took place by a blinded observer using the recorded videotapes. The level of anxiety in the child was assessed as the video was paused at four points: following the dental examination, administration of LA, completion of pulpotomy procedures, and after fitting of SSCs (Table 1). (**3) Subjective parameter**: utilizing a modified Facial Affective Scale (FAS) based on Quiles et al. [25] Anxiety was subjectively recorded with it. It has three basic, colored faces that represent, respectively, (a) no anxiety, (b) some anxiety, and (c) very high anxiety. Every child was asked to select the face that best reflected the feeling that he was experiencing. Assessments were carried out before and following the procedure. (Fig. 3)

**Table 1:** The venham clinical anxiety scale (VCAS)

Rating	Anxiety rating scale
0.	Relaxed, smiling, willing, and able to converse.
1.	Uneasy, concerned. Protest briefly and quietly to indicate discomfort; hands remain down or partially raised to signal discomfort, and The facial expression is tense and capable of cooperating.
2.	Tense: tone of voice, questions, and answers reflect anxiety. During stressful procedures, verbal protest, (quiet) crying, hands tense and raised (not interfering much may touch dentist’s hand or instrument, but not pull at it). The child still complies with the request to cooperate.
3.	Reluctant: pronounced verbal protest, crying, Using hands to stop procedure. Treatment proceeds with difficulty.
4.	Interference: General crying is not related to treatment. More prominent body movement sometimes requires physical restraint, and protests disrupt the procedure.
5.	Out of contact. Hard, loud crying, unable to listen, trying to escape. Physical restraint is required.

### Examiner reliability

A single dentist performed all procedures, and a blinded observer scored video recordings of the children’s behavior using the VCAS. The observer then re-evaluated the videos after a one-week interval to assess consistency. The resulting Kappa scores (0.70-1.00) indicated good reliability for the VCAS in measuring children’s dental anxiety.

### Statistical analysis

The study employed a combination of statistical tests to analyze the collected data. Shapiro-Wilk tests and Q-Q plots assessed the normality of (age and heart rate) data, allowing for presentation using means and standard deviations. The non-normally distributed Venham scale data was presented with medians, interquartile ranges, minimums, and maximums. Descriptive statistics like frequencies and percentages summarized qualitative data (gender, hearing impairment severity, Frankl behavior scale, and Facial Affect Scale). ***Independent t-test*** was used to analyze age and heart rate between groups, while ***Pearson’s Chi-square test*** analyzed differences in qualitative variables. ***Repeated measures ANOVA*** evaluated the impact of procedures on heart rate, and the ***Mann-Whitney U test*** compared Venham scale scores. Finally, the ***Friedman test***, potentially followed by ***Bonferroni-corrected post-hoc tests***, explored variations in Venham scale scores across different time points in case of significant results. All analyses were two-tailed with a significance level of *p* ≤ 0.05. The data analysis was conducted using IBM SPSS version 23 for Windows, Armonk.

## Results

**Fig. 4:**
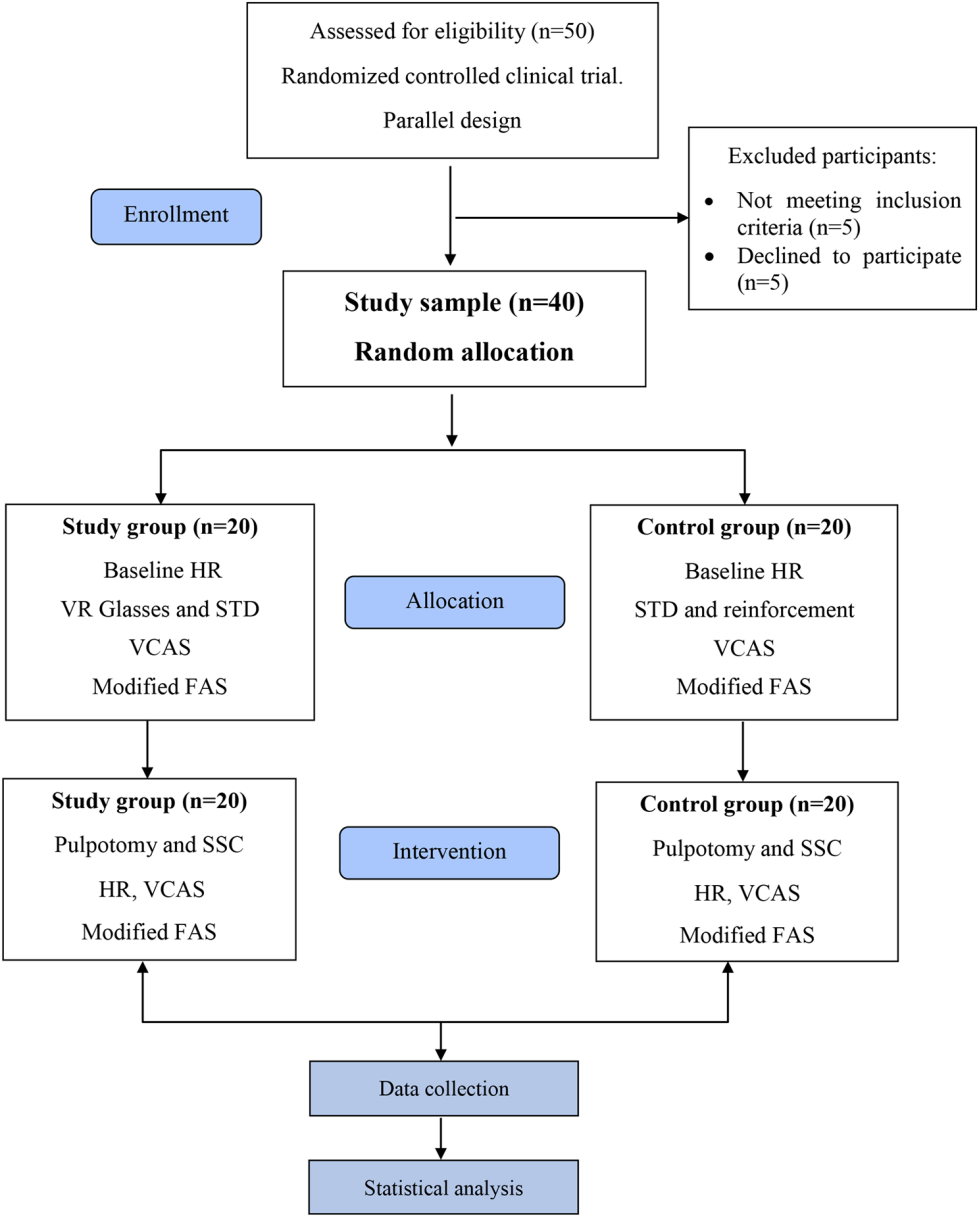
CONSORT flow chart

Fifty patients were examined in this study. Five patients declined to participate. Five more did not meet the requirements for participation. Throughout the entire time, there were neither patient dropouts nor incomplete data. (Fig. 4). Patient recruitment was initiated on the first day of October 2023 and continued through the final day of February 2024. A total of 40 children participated in the study, 20 of which were assigned to the VR test group (Group I) and 20 to the conventional behavior technique control group (Group II). The mean age of children assigned to group I was 6.43 (± 0.67), whereas those assigned to group II was 6.15 (± 0.93) with no statistically significant difference (*p* = 0.292). Ten patients were males (50%), and 10 were females (50%) in both the study and control groups (*p* = 1.00). Considering the extent of hearing impairment between the two groups, in group I, 9 patients (45%) were moderate, and 11 patients (55%) were severe, whereas, in group II, 7 patients (35%) were moderate and 13 (65%) were severe with no statistically significant differences between them (*p* = 0.744). 35% of the children in group I showed negative behavior (Frankl rating 2), and 65% showed positive behavior (Frankl rating 3). In group II, 50% of children showed negative behavior, and 50% showed positive behavior, with no statistically significant difference between the two groups *(p* = 0.337). (Table 2)

**Table 2:** Baseline characteristics of the whole sample

		Study group (group I ) (*n* = 20)	Control group (group II) (*n* = 20)	*p*-value
**Age: Mean ± SD**		6.43 ± 0.67	6.15 ± 0.93	0.292
**Gender: n (%)**	**Male**	10(50%)	10 (50%)	1.00
	**Female**	10 (50%)	10 (50%)	
**Frankl Behavior**	**Negative**	7 (35%)	10 (50%)	0.337
	**Positive**	13 (65%)	10 (50%)	
**Severity of hearing disability: n (%)**	**Moderate**	9 (45%)	7 (35%)	0.744
	**Severe**	11 (55%)	13 (65%)	

### The results of *heart rate readings*

The mean HR did not significantly differ between the study and control groups at baseline or during different dental operations: At baseline in group I, it was (101.05 ± 3.12), and in group II, it was (102.00 ± 4.03 ) (*p* = 0.409); during injection, the mean HR in group I was (110.20 ± 9.15), and in group II was(111.30 ± 6.20)( *p* = 0.659), during pulpotomy the mean HR in group I was (106.75 ± 5.68) and in group II was (107.95 ± 4.98) (*p* = 0.482), and during SSC preparation the mean HR in group I was (102.25 ± 3.31) and group II was (103.85 ± 2.46) (*p* = 0.090) presented in Table 3. However, Repeated-measures ANOVA presented in Table 3 revealed that HR changed significantly within each group throughout different procedures in the same session (*p* = 0.0001).

**Table 3:** Comparison of mean heart rate between study and control groups at different procedures

Procedures	Study group (group I ) (*n* = 20)	Control group (group II) (*n* = 20)	*p*-value
	Mean ± SD		
Baseline	101.05 ± 3.12	102.00 ± 4.03	0.409
Local anesthesia	110.20 ± 9.15	111.30 ± 6.20	0.659
Pulpotomy	106.75 ± 5.68	107.95 ± 4.98	0.482
SSC	102.25 ± 3.31	103.85 ± 2.46	0.090
*p-value*	< 0.0001*	< 0.0001*	

Statistically significant difference at p value < 0.05

The pairwise comparison of the mean HR (Table 4) showed that in group I, according to the baseline assessment, there was a statistically significant HR increase following the LA administration (*p* = 0.001). The pulpotomy procedure further elevated HR, demonstrating a statistically significant difference compared to baseline (*p* = 0.002). However, no significant difference in mean HR was observed between baseline and SSC preparation (*p* = 1.00). Compared to LA administration, both pulpotomy and SSC preparation resulted in significantly lower HR levels (*p* = 0.031 and *p* = 0.002, respectively).

**Table 4:** Pairwise comparison of heart rate between different procedures within each group

Procedures	Compared to	*p*-value	
		Study group(group I )	Control group(group II)
**Baseline**	Local anesthesia	0.001*	< 0.0001*
	Pulpotomy	0.002*	0.001*
	SSC	1.00	0.485
**Local anesthesia**	Pulpotomy	0.031*	0.006*
	SSC	0.002*	< 0.0001*
**Pulpotomy**	SSC	0.004*	< 0.0001*

*Statistically significant difference at p value ≤ 0.05

In group II, compared to the baseline assessment, the administration of LA and the pulpotomy procedure resulted in a statistically significant increase in HR (*p* < 0.0001 and *p* = 0.001, respectively). However, no significant difference in mean HR was observed between baseline and SSC preparation *(p* = 0.485). Compared to LA administration, pulpotomy and SSC preparation demonstrated significantly lower HR levels (*p* = 0.006 and *p* < 0.0001).

### Anxiety scores measured by VCAS rating assessment

At baseline, there was not a significant difference in either group’s anxiety scores (*p* = 0.739); similarly, during LA administration (*p* = 0.282), a pulpotomy (*p* = 0.451), and during SSC preparation (*p* = 0.284). However, according to the Friedman test, both groups experienced changes in anxiety throughout different dental procedures, regardless of the technique used (*p* < 0.0001) (Table 5). The pairwise comparison of dental anxiety between different procedures within each group (Table 6) revealed that In group I, there was a significant increase in the anxiety level during LA compared to baseline (*p* = 0.003). There was a statistically significant decrease in anxiety levels during SSC compared to LA (*p* = 0.016). There was no statistical difference in anxiety level between the baseline and pulpotomy (*p* = 0.193) nor between baseline and SSC preparation (*p* = 1.00). No statistical significance was also noted between the pulpotomy procedure and SSC preparation (*p* = 0.589).

**Table 5:** The venham clinical anxiety scale assessment of anxiety levels at various stages

Procedures	Study group (group I ) (*n* = 20)			Control group (group II) (*n* = 20)			*p*-value
	Mean ± SD	Median (IQR)	Min - Max	Mean ± SD	Median (IQR)	Min - Max	
Before	0.30 ± 0.47	0.00 (1.00)	0.00–1.00	0.35 ± 0.49	0.00 (1.00)	0.00–1.00	0.739
Local anesthesia	1.05 ± 0.69	1.00 (1.00)	0.00–2.00	1.35 ± 0.99	1.50 (2.00)	0.00–3.00	0.282
Pulpotomy	0.75 ± 0.55	1.00 (1.00)	0.00–2.00	0.95 ± 0.83	1.00 (2.00)	0.00–2.00	0.451
SSC	0.40 ± 0.60	0.00 (1.00)	0.00–2.00	0.55 ± 0.51	1.00 (1.00)	0.00–1.00	0.284
*p-value*	< 0.0001			< 0.0001			

*Statistically significant difference at p value < 0.05

**Table 6:** Pairwise comparison of Venham scale between different procedures within each group

Procedures	Compared to	*p*-value	
		Study group(group I )	Control group(group II)
Before	Local anesthesia	0.003*	0.001*
	Pulpotomy	0.193	0.165
	SSC	1.00	1.00
Local anesthesia	Pulpotomy	1.00	0.755
	SSC	0.016*	0.013*
Pulpotomy	SSC	0.589	0.755

*Statistically significant difference at p value ≤ 0.05

In group II, there was also a significant increase in anxiety during LA compared to baseline (*p* = 0.001) and a statistically significant decrease in anxiety during SSC preparation compared to LA (*p* = 0.013). There was no statistical difference in anxiety level between the baseline and pulpotomy (*p* = 0.165) nor between baseline and SSC preparation (*p* = 1.00). No statistical significance was also noted between the pulpotomy procedure and SSC preparation (*p* = 0.755).

### Anxiety measurements using modified FAS

The baseline anxiety levels of the patients did not have any statistically significant difference between the study group (14 no anxiety, 6 highly anxious) and the control group (12 no anxiety, 8 highly anxious) (*p* = 0.507) or after treatment: study group: (12 no anxiety, 8 highly anxious) and control group: (11 no anxiety, 9 highly anxious), (*p* = 0.749). (Table 7)

**Table 7:** Comparison of anxiety level using a modified facial affective scale (FAS) between groups

		Study group(group I ) (*n* = 20)	Control group(group II) (*n* = 20)	*p*-value
**Before procedure: ** **n (%)**	No anxiety	14 (70%)	12 (60%)	0.507
	Some anxiety	0 (0%)	0 (0%)	
	Very high anxiety	6 (30%)	8 (40%)	
**After procedure: ** **n (%)**	No anxiety	12 (60%)	11 (55%)	0.749
	Some anxiety	0 (0%)	0 (0%)	
	Very high anxiety	8 (40%)	9 (45%)	

*Statistically significant difference at p value ≤ 0.05

## Discussion

Children with moderate to severe HI comprise a group known for higher dental anxiety than normal children due to the difficulty in communication, resulting in social fears, fear of potential injury, and fear of the unfamiliar environment [26]. Few literature research addressed different techniques for managing these children on the dental chair. Recognizing the high prevalence of dental anxiety among HI children, documented in studies like Suhani et al. [27] and Saatchi et al. [28], revealed high negative behavior (Frankl rating 2), reaching 42% of the sample.

VR technology has proved effective in reducing anxiety and fear in normal children [29], but little is known regarding its effectiveness in managing children with HI. Therefore, this study assessed the effect of employing virtual reality glasses that showed cartoons in sign language as a distraction method during pulpotomy treatment to lessen dental anxiety in children with moderate to severe HI. A multifaceted approach was implemented, combining VR technology and the STD method. By simultaneously providing effective communication and engaging distractions, the aim was to familiarize children with HI regarding dental settings and to distract them from the dental procedure.

The 5-to-7-year-old age range was selected for this study because dental anxiety decreases with age. Consequently, older children than this age group, possessing greater cognitive strength and enhanced environmental awareness, are likely to exhibit lower levels of dental anxiety [30]. Meanwhile, children below five years may encounter great difficulty in communication due to limited cognitive development and social fear [31].

The study involved children with moderate to severe HI, as mild HI may not have any communication problems and can usually be treated like normal children [32]. Children with previous bad dental experiences or with Frankl behavior rating 1 were excluded, as these children will usually need other advanced behavior management techniques like sedation or general anesthesia [33].

It is advisable to select a minimum of two anxiety measures to conduct behavioural research, particularly in young children, because their cognitive, emotional, and social development is more limited than that of adults; moreover, since children with HI have difficulty with hearing, which is a key sense, the study used a combination of 3 methods to assess their anxiety for accuracy purpose. Heart rate was measured as a physiological technique to evaluate pain, as it is one of the most precise indicators of the autonomic reaction to pain stimuli [34]. It removes the possibility of bias from the observer and the children’s subjective self-reporting. Additionally, the VCAS objective scale was used as it has been proven to be an effective tool in measuring anxiety by many researchers [35]. The subjective measurement of anxiety was assessed by adapting the FAS from Quiles et al. [25] to enhance the simplicity and interpretability of the scale. This improved the child’s response and reduced confusion.

The results of the present study showed that there was no statistically significant difference in dental anxiety levels between the two groups, as indicated by the three parameters of anxiety used. Thus, VR glasses did not decrease child anxiety during dental procedures. These results were consistent with those of Fakhruddin et al. [12] who discovered that children receiving VR eyewear had significantly greater levels of anxiousness. This might be due to the complete visual obstruction caused by the VR glasses, potentially inducing anxiety in the unfamiliar and potentially frightening dental environment. Moreover, it is well known that children with HI often need to use their sight more to understand communication, like lip-reading, and to discover all their surroundings to feel secure in a new environment like the dental session.

On the other hand, Varshitha et al.‘s [11] findings revealed that the VR glasses study group showed a significant decrease in anxiety than the control group. The difference in the results between the present study and their study can be attributed to the different age groups as they included older age groups ranging from 6–11-year-old children with more developed cognitive, emotional, and social abilities. Children at that age could remain diligent at a stressful task with longer attention spans and more liable for enduring unpleasant situations; moreover, in their study, they only conducted a noninvasive dental procedure, while the pulpotomy procedure was carried out in the current research.

Post hoc analysis of the study group and the control group showed a rise in anxiety levels during the LA administration. This suggests that the injection pain might have been strong enough to overcome the distracting effect of the VR glasses. Similar findings were reported in a study by Suhani et al. [27] and also Felemban et al. [36]. This could be attributed to the fact that some parents use the idea of the threat of injection as a means of managing children’s behavior, bearing in mind that injection itself is an invasive and fear-provoking procedure. Anxiety then gradually decreased as the anesthesia started to become effective during pulpotomy and SSC preparation, and the child became more relaxed.

During the clinical trial, we observed that the children in the study group using VR glasses attempted to remove them throughout the session. They would look at the dentist’s actions and then put the VR glasses back on after consulting with their guardian. The sign language cartoon employed for distraction did not capture the children’s attention effectively. Their focus remained on observing the surrounding environment rather than engaging with the cartoon’s content. The HI children’s reliance on visual cues for communication, including lip-reading, suggested that clear and consistent communication throughout the dental procedure might be a more impactful approach for reducing anxiety.

The limitation of this study was the inability to blind the operator or the external investigator from the child’s use of the VR. Additionally, the VR glasses used in this study were slightly large for some children, potentially impacting their comfort during dental procedures. Furthermore, the size of the VR glasses made it more challenging to apply a rubber dam. Finally, a sub-analysis between children with positive behavior and those with negative behavior groups should have been compared and included in the results section, also considered a study limitation.

The study confirmed the null hypothesis as there was no significant difference in the reduction of dental anxiety in children with HI between the VR glasses and the traditional technique of child behavior management.

## Conclusion

From the results of the present study, it can be concluded that the effect of VR glasses did not reduce anxiety in children with HI and was comparable to conventional behavior management techniques during the whole dental procedure.

### Why this article is important

Pediatric dentists should be aware of communication strategies and procedural modifications for children with HI, which might be more crucial for managing dental anxiety than the implementation of VR distraction techniques in its current form.

Pediatric dentists should prioritize the exploration and implementation of novel technologies and treatment modalities specifically designed to manage anxiety in children with disabilities.

## Electronic supplementary material

Below is the link to the electronic supplementary material.

Supplementary Material 1

## Data Availability

Upon a reasonable request, the corresponding author may make the study’s dataset available. Due to restrictions (such as the fact that they contain information that can risk research participants’ privacy), the data are not publicly available.

## Abbreviations

3D: Three dimensional
ASA: American Society of Anesthesiologists
AAPD: American Academy of Pediatric Dentistry
VR: Virtual reality
SSC: Stainless steel crown
FAS: Facial affective scale
STD: SHOW- TELL – DO
HI: Hearing Impairment
VCAS: Venham clinical anxiety scale
LA: Local anesthesia
HR: Heart rate
